# Less Vertebral Bone Mass after Treatment with Macitentan in Mice: A Pilot Study

**DOI:** 10.1155/2019/2075968

**Published:** 2019-02-19

**Authors:** Zhong-Yu Liu, Man-Ting Au, Tian-Wei He, Bu Yang, Bin Liu, Liang-Ming Zhang, Chun-Xiao Luo, Li-Min Rong, Chun-Yi Wen

**Affiliations:** ^1^Department of Spine Surgery, Institute of Drug Clinical Trial for Orthopedic Diseases, The Third Affiliated Hospital of Sun Yat-Sen University, Guangzhou, China; ^2^Interdisciplinary Division of Biomedical Engineering, Faculty of Engineering, The Hong Kong Polytechnic University, HKSAR, China

## Abstract

**Purpose:**

Blood vessels and skeleton interact together. Endothelin-1 is a potent vasoconstrictor and also has an effect on bone metabolism. The dual antagonist to both endothelin-1 type A and B receptors, Macitentan, has been approved for clinical management of pulmonary arterial hypertension while little is known about the secondary effect of the drug on spine. We aimed to answer how vertebral bone mass responded to Macitentan treatment in mice.

**Methods:**

Sixteen male balb/c mice at 6 months were randomly assigned into 2 groups. Vehicle and Macitentan were administrated via intraperitoneal injection to Control group and Treatment group, respectively, for 4 months. At sacrifice, plasma endothelin-1 was evaluated with ELISA and vertebral bone mass was evaluated with Microcomputed Tomography and histological analysis.

**Results:**

We found higher plasma endothelin-1 level (p<0.01) and less vertebral bone mass (p<0.05) in Treatment group compared to controls. Moreover, less osteoblasts and more osteoclasts were observed in the vertebral trabecular bone in the Treatment group compared to controls, by immunohistochemistry of the cell-specific markers.

**Conclusions:**

Treatment with Macitentan is associated with significant lower vertebral bone mass and therefore the secondary effect of dual antagonists to endothelin-1 receptors on the skeleton should be monitored and investigated in clinical practice. Both osteoblasts and osteoclasts may be involved while the molecular mechanism needs to be further explored.

## 1. Introduction

Blood vessels and the skeleton are closely connected [1]. Vascular diseases and bone remodeling disorders (e.g., osteoporosis, osteoarthritis) may share common biological mechanisms [2], including dysfunction of OPG/RANK/RANKL system [3, 4], altered PTH level [5, 6], and aberrant WNT [7] and BMP signaling pathways [8–11]. Additionally, human mesenchymal stem cells (hMSCs), including the newly identified human skeletal stem cells (hSSCs) [12] that give rise to the skeleton, are derived from perivascular cells [13]. Therefore, vasoactive molecules might also have an effect on the skeleton.

Endothelin-1 (ET-1), a peptide predominantly secreted by the vascular endothelial cells, is a potent vasoconstrictor [14] and also plays an important role in the regulation of postnatal bone remodeling [15]. ET-1 has two receptors, endothelin type A receptor (ETAR) and type B receptor (ETBR). The dual antagonist to both ETAR and ETBR, Macitentan, has been approved for clinical management of pulmonary arterial hypertension (PAH) [16]; the secondary effect of the drug on vertebral bone mass is of great interest but still not reported.

In this* in vivo* study, we demonstrated the effect of Macitentan on mice vertebral bone mass with Microcomputed Tomography (*μ*CT) and histology. Preliminary evaluation of the osteoblasts and osteoclasts was also performed by immunostaining of the cell-specific markers.

## 2. Materials and Methods

### 2.1. Animals

All the animal experiments and procedures were in accordance with the guidelines for the use and care of laboratory animals. Sixteen male (not female because of the possible influence of estrogen fluctuation on bone mass due to menstruation and/or menopause) Balb/c mice at the age of 6 months were obtained for the study. They weighed between 24 and 26 grams and were housed in standard plastic cages (one mouse per cage) on sawdust bedding in an air-conditioned room at 21±0.4°C and 47±1% humidity under 12-hour light/12-hour dark cycle. All animals were fed standard chow and had free access to water.

### 2.2. Drugs and Chemicals

ETAR/BR dual antagonist—Macitentan—was obtained from Actelion Pharmaceuticals (Allschwil, Switzerland). Ketamine and xylazine for anesthesia were purchased from IE Ulagay (A.S. Istanbul, Turkey), and Penicillin was obtained from Sanofi-Aventis (Paris, France). Except for those specially stated, all the other chemicals for the laboratory experiments were purchased from Merck (Darmstadt, Germany).

### 2.3. Grouping

The 16 mice were randomly assigned into two groups. Beginning from the first day, we daily administrated Macitentan (10mg/kg B.W.) dissolved in vehicle (MEM-alpha with 10%DMSO, 1%Penicillin and 15%Fetal Bovine Serum) to the Treatment group and the same amount of vehicle alone to Control group via intraperitoneal injection. Mice were sacrificed at 4 months by exsanguination under general anesthesia for tissue collection.

### 2.4. ELISA of Plasma ET-1

For comparison of plasma ET-1 between groups, blood samples (about 1ml) were drawn at the time of sacrifice from the left ventricle under general anesthesia using ketamine/xylazine/normal saline cocktail (1ml:0.5ml:8.5ml, 1ml/100g B.W.). Plasma ET-1 level was evaluated using ELISA kits (ab133030, Abcam, Cambridge, UK) according to the manufacturer's instructions.

### 2.5. Microcomputed Tomography (*μ*CT) of the 5^th^ Lumbar Vertebra

After sacrifice, the 5^th^ lumbar vertebrae (the last but one lumbar vertebra caudally) of the mice were scanned with a quantitative *μ*CT system (Viva CT40, Scanco, Switzerland).

Isotropic voxel size for the scans was 10.5*μ*m. X-ray voltage of 70kV and 1.0 filter were applied. After standardized reconstruction by a modified Feldkamp algorithm via SkyScan recon software, the data sets for each vertebra were analyzed using SkyScan CT-analyzer software. Semiautomated contouring was used to select a region of interest (ROI) comprising all the trabecular bone in the whole vertebral body. The microarchitecture of the vertebra was examined with ANT™ software (SkyScan). The three-dimensional structure and morphometry was constructed and analyzed for BV/TV (%): trabecular bone volume per tissue volume, Tb.N. (mm^−1^): trabecular number, Tb.Th. (mm): trabecular thickness, and Tb.Sp. (mm): trabecular separation.

### 2.6. Histology and Histomorphometry

At the time of sacrifice, we resected and fixed the 5^th^ lumbar vertebrae in 10% buffered formalin for 72 h (during which *μ*CT scan was performed), decalcified them in 10% EDTA (pH 7.4) for 20 days at room temperature, and embedded them in paraffin (Leica biosystems, Nussloch, Germany). Three micrometer thick coronal-oriented sections of the 5^th^ lumbar vertebra were processed for Hematoxylin & Eosin (H&E) staining. Images were captured using Nikon H600L Microscope and Image-Pro Plus version 5.0 (Media Cybernetics, Rockville, USA). As quantitative analysis of the bone mass could be better achieved by *μ*CT, only descriptive analysis was performed on the H&E slides.

### 2.7. Immunohistochemistry and TRAP Staining

To evaluate osteoblasts, immunostaining was performed using a standard protocol [17]. We incubated sections with primary antibodies to mouse Alkaline phosphatase (ALP, PA1004, Boster, Pleasanton, USA) and Osteocalcin (OCN, ab93876, Abcam, Cambridge, UK) overnight at 4°C. A biotinylated horseradish peroxidase detection system (Vectastain, PK-6200, Vector Laboratories, Burlingame, USA) was subsequently used to detect the immunoactivity, followed by incubation in 3,3'-diaminobenzidine (DAB, SK-4100, Vector Laboratories, Burlingame, USA) and counterstaining with hematoxylin. Also, tartrate-resistant acid phosphatase (TRAP) staining was performed for osteoclasts. Descriptive analysis to the immunostaining was performed by comparing the number of cells in the view field that are positive with the markers mentioned above. At least three mice per group were examined. Three equidistant sections spaced at 200 *μ*m apart throughout the middle 1/3 coronal section of the vertebra were evaluated.

### 2.8. Statistical Analysis

All results were presented as the mean ± standard deviation (SD). The data between Treatment and Control groups were compared using Student's t test. The level of significance was set at p < 0.05. IBM SPSS v.21 software was used for data analyses.

## 3. Results

One mouse in the Control group died of tumor. Therefore the final sample size of the Control group was 7 and the Treatment group was 8 for quantitative analysis.

### 3.1. Plasma ET-1 in Control and Treatment Groups

**Figure 1:**
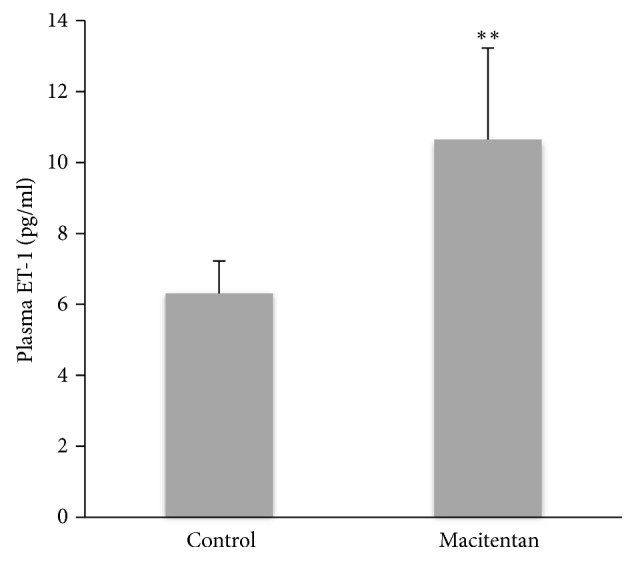
*Plasma ET-1 level in different groups* ELISA revealed that plasma ET-1 level was significantly higher in Macitentan Treatment group compared to Control group (*∗∗*p<0.01, n=7 for Control group, and n=8 for Treatment group).

Quantitative analysis of ELISA revealed a significant higher plasma ET-1 in Treatment group compared to controls (p<0.01) at 4 months (Figure 1).

### 3.2. Vertebral Bone Mass in Control and Treatment Groups

**Figure 2:**
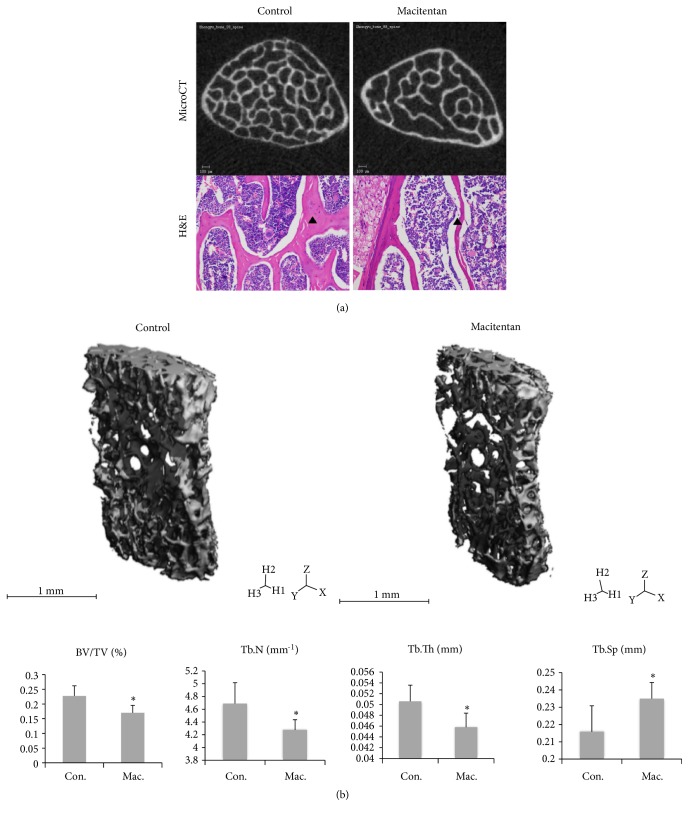
*Vertebral bone mass in different groups* (a) MicroCT images of the transverse plane and H&E staining of the coronal sections of the 5^th^ lumbar vertebral body showed fewer and thinner trabeculae (black triangle) in Treatment group compared to controls. (b) Quantitative analysis revealed significant lower BV/TV, Tb.N, and Tb.Th and higher Tb.Sp in Treatment group compared to controls (*∗*p<0.05, n=7 for Control group, n=8 for Treatment group. Con.: Control, Mac.: Macitentan, BV/TV: trabecular bone volume per tissue volume, Tb.N.: trabecular number, Tb.Th.: trabecular thickness, and Tb.Sp.: trabecular separation).

MicroCT demonstrated a significant lower bone mass in Treatment group as indicated by BV/TV, Tb.N, Tb.Th, and Tb.Sp compared to Control group (all p<0.05). The bone mass under histological observation was consistent with *μ*CT findings (Figure 2).

### 3.3. Osteoblasts and Osteoclasts in Control and Treatment Groups

**Figure 3:**
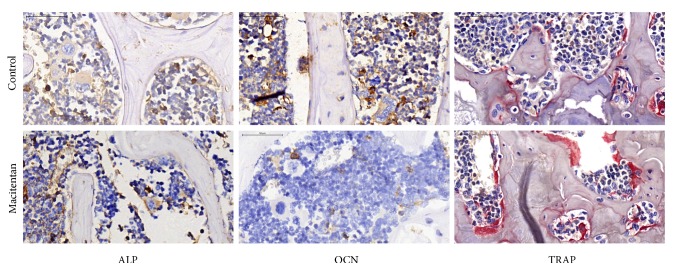
*ALP, OCN, and TRAP expression in the 5* ^*th*^ *lumbar vertebral spongiosa* Immunohistochemistry demonstrated fewer ALP(+) and OCN(+) cells (brown) but more TRAP(+) (red) cells in Treatment group compared to Control group (ALP: alkaline phosphatase, OCN: Osteocalcin, and TRAP: tartrate-resistant acid phosphatase).

We found less ALP and OCN but more TRAP expression in the 5^th^ lumbar vertebral spongiosa, indicating fewer osteoblasts but more osteoclasts, in Treatment group compared to Control group at 4 months (Figure 3).

## 4. Discussion

In this study, we tested the effect of the anti-PAH drug—Macitentan on vertebral bone mass. We found significant lower vertebral bone mass in the Treatment group compared to controls at 4 months. The decreased bone mass was associated with and might result from the decreased osteoblast activity as well as the increased osteoclast activity.

ET-1 is a vasoconstrictor [14] substantially involved in the pathophysiology of multiple vascular diseases [18–20]. Meanwhile, its role in bone remodeling is also drawing much attention [15]. Targeted inactivation of ETAR in mature osteoblasts induced lower tibial trabecular bone volume in vivo [21] and global ET-1 knockout mice had severe hypoplasia in craniofacial bones [22]. Also, ET-1 was reported to enhance osteogenesis of bone marrow-derived mesenchymal stem cells (BMSCs) [23, 24]. The results of previous studies indicated a positive role of ET-1 in bone formation. Consistently, we demonstrated that blockade of ET-1 signaling pathway resulted in low bone mass. Our findings suggested the potential adverse effect of the dual antagonists to endothelin receptors (ETRs) on the skeleton and has built a translational bridge from previous fundamental researches to further clinical investigations. Therefore, bone mass of PAH patients taking these drugs should be closely monitored to avoid progressive bone loss and subsequent osteoporotic fractures.

Clinical observations demonstrated that postmenopausal osteoporotic women presented higher serum level of ET-1 [25], suggesting that the status of low bone mass was accompanied by systemic overexpression of ET-1. Accordingly, we also found a dramatic increase of plasma ET-1 in the Treatment group with low bone mass, which could be explained by the mechanism of compensatory ET-1 secretion due to ETRs blockade.

Some limitations should be mentioned in our current research. First, dual antagonists to ETRs were usually administrated* perorally* in clinical practice. However, in order to standardize the drug dose between individuals, intraperitoneal rather than oral administration of the drug was performed in our study. Additionally, Macitentan could merely dissolve in natural saline or PBS alone and DMSO was needed as the cosolvent but was toxic. We found that MEM-alpha with Fetal Bovine Serum was a good solvent for Macitentan with the lowest concentration (10%) of DMSO. The solvent turned out feasible with low toxicity as only one mouse died of tumor rather than the toxic effect of DMSO during the experiment. Next, due to cost limitation, we only tried the 10mg/kg body weight dose of Macitentan in this pilot study, according to a previously published research in which Macitentan was given to mice by a peritoneal catheter at 0.1, 1, and 10mg/kg body weight per day for 5 weeks and biological effect was found at 10mg/kg [26]. Dose-dependent effect of the drug is an important question that needs to be addressed in forthcoming studies. Last but not least, the number and function of osteoblasts and osteoclasts in different groups could not be precisely evaluated in vivo. The possible involvement of osteoblasts/osteoclasts and the underlying molecular mechanism is to be further explored by experiments at cellular and molecular levels.

In conclusion, treatment with Macitentan is associated with significant lower vertebral bone mass in mice and therefore the secondary effect of dual antagonists to ETRs on the skeleton should be monitored and investigated in clinical practice. Both osteoblasts and osteoclasts seem to be involved while the molecular mechanism needs to be further explored.

## Data Availability

All data generated and analyzed during the current study are available from the corresponding author on reasonable request.
